# A novel immune checkpoints-based signature to predict prognosis and response to immunotherapy in lung adenocarcinoma

**DOI:** 10.1186/s12967-022-03520-6

**Published:** 2022-07-25

**Authors:** Nan Sun, Yuejun Luo, Bo Zheng, Zhihui Zhang, Chaoqi Zhang, Zhen Zhang, Guochao Zhang, Fengwei Tan, Qi Xue, Shugeng Gao, Jie He

**Affiliations:** 1 Department of Thoracic Surgery, National Cancer Center/National Clinical Research Center for Cancer/Cancer Hospital, Chinese Academy of Medical Sciences and Peking Union Medical College, Beijing, 100021 China; 2 State Key Laboratory of Molecular Oncology, National Cancer Center/National Clinical Research Center for Cancer/Cancer Hospital, Chinese Academy of Medical Sciences and Peking Union Medical College, Beijing, China; 3 Department of Pathology, National Cancer Center/National Clinical Research Center for Cancer/Cancer Hospital, Chinese Academy of Medical Sciences and Peking Union Medical College, Beijing, 100021 China; 4 Biotherapy Center, the First Affiliated Hospital of Zhengzhou University, Zhengzhou, 450052 Henan China

**Keywords:** Lung adenocarcinoma, Immune checkpoints, Prognosis, PD-L1, Immunotherapy

## Abstract

**Background:**

Except for B7-CD28 family members, more novel immune checkpoints are being discovered. They are closely associated with tumor immune microenvironment and regulate the function of many immune cells. Various cancer therapeutic studies targeting these novel immune checkpoints are currently in full swing. However, studies concerning novel immune checkpoints phenotypes and clinical significance in lung adenocarcinoma (LUAD) are still limited.

**Methods:**

We enrolled 1883 LUAD cases from nine different cohorts. The samples from The Cancer Genome Atlas (TCGA) were used as a training set, whereas seven microarray data cohorts and an independent cohort with 102 qPCR data were used for validation. The immune profiles and potential mechanism of the system were also explored.

**Results:**

After univariate Cox proportional hazards regression and stepwise multivariable Cox analysis, a novel immune checkpoints-based system (LTA, CD160, and CD40LG) were identified from the training set, which significantly stratified patients into high- and low-risk groups with different survivals. Furthermore, this system has been well validated in different clinical subgroups and multiple validation cohorts. It also acted as an independent prognostic factor for patients with LAUD in different cohorts. Further exploration suggested that high-risk patients exhibited distinctive immune cells infiltration and suffered an immunosuppressive state. Additionally, this system is closely linked to various classical immunotherapy biomarkers.

**Conclusion:**

we constructed a novel immune checkpoints-based system for LUAD, which predicts prognosis and immunotherapeutic implications. We believe that these findings will not only aid in clinical management but will also shed some light on screening appropriate patients for immunotherapy.

**Supplementary Information:**

The online version contains supplementary material available at 10.1186/s12967-022-03520-6.

## Introduction

Lung adenocarcinoma (LUAD) is the most common variant of non–small cell lung cancer (NSCLC) across the world. LUAD has become significantly more prevalent over the past two decades, with a similar decrease in squamous cell carcinoma during the same period [1]. There have been advances in the current strategy of combining various therapies with tyrosine kinase inhibitors (TKIs) to treat patients with LAUD. However, the 5-year overall survival (OS) rate for patients with LUAD remains poor [2, 3]. At present, there is an urgent need to identify specific prognostic biomarkers in patients with LAUD, to enable the development of optimized, appropriate treatment protocols and clinical management for patients with different elements of risk. Several studies have attempted to predict and evaluate prognosis in patients with LAUD, through diverse gene expression profiles and bioinformatics approaches [4–6]**.** However, these studies usually incorporate genes from the entire genome or transcriptome and do not consider the intrinsic biological functions. This may give rise to the fact that many of these signatures remain merely mathematical but unable to truly reflect the real characters inherent in the tumor.

In recent years, the treatment of LUAD has developed rapidly. In addition to traditional surgery, radiotherapy, and chemotherapy, molecular targeted therapy, novel strategy like immunotherapy has been becoming of increasingly great concern [7, 8]. The introduction of immune checkpoint-blocking antibodies that target programmed death 1 (PD-1) receptor as well as against its ligand, programmed death-ligand 1 (PD-L1) of the B7-CD28 family has significantly improved survival rates in patients with advanced lung cancer. These antibodies are available for clinical use [9–11]. However, beneficial immunotherapeutic effects were only observed in a subgroup of patients with response rates of 17–21% [12], suggesting that there may be other immune checkpoints in the LAUD tumor microenvironment (TME).

In addition to these star targets of the B7-CD28 family, several emerging and promising immune checkpoints have been identified [13]. Most of these novel immune checkpoints are from the TNF superfamily, including the TNF ligand superfamily (TNFSF) as well as the TNF receptor superfamily (TNFRSF), which are responsible for regulating pathways related to cell survival, differentiation, and non-immunogenic death. Most notably, these novel immune checkpoints belonging to the TNF superfamily also play a critical function in regulating the immune system, providing co-stimulatory or co-inhibitory signals vital for natural and adaptive immunity with an emphasis on T-cell responsiveness [14, 15].

These novel immune checkpoints molecules can trigger inflammatory activities in several cells in the TME, including cells like T and B lymphocytes as well as tissue-resident cells such as epithelial and fibroblasts cells [16]. Therefore, modulating and controlling the interaction of these novel immune checkpoints would be a novel therapeutic target with great potential for tumor treatment. Meanwhile, many cancer therapies targeting these novel immune checkpoints are in full swing, including preclinical and clinical trials. For example, therapies targeting OX40, CD40, and CD27 have achieved favorable progress in various tumor treatments, including for patients with lung cancer [14, 17, 18]. This observation inspired us to explore and establish a novel immune checkpoints-based prognosis system for LUAD, revealing immune features for patients with LUAD.

Herein, we firstly enrolled 1883 LUAD samples from nine independent cohorts, including eight public cohorts and an independent cohort. Then, we systematically explored these novel immune checkpoints in LAUD and filtered out genes with the most prognostic value. From this, we constructed a signature based on the combination of LTA, CD160, and CD40LG, which was also well-validated in different cohorts. Finally, we further examined the clinical characteristics, immunotherapy responses, and immune cells infiltration of the prognostic signature. The novel combination of LTA, CD160, and CD40LG, may help us understand the immune status of patients with LUAD, but also optimize available tumor immunotherapies.

## Materials and methods

### Publicly available mRNA expression datasets

We collected 1731 publicly available samples from The Cancer Genome Atlas (TCGA), including level three RNA-seq expression data from 502 LAUD cases (Illumina HiSeq 2000). All samples had corresponding prognostic data and other clinical information. These data were downloaded from the Cancer Genomics Browser of the University of California Santa Cruz (UCSC) (https://genomecancer.ucsc.edu) and served as the training cohort. The microarray data and corresponding survival information for the remaining 1670 samples were gathered from seven different Gene Expression Omnibus (GEO) datasets (http://www.ncbi.nlm.nih.gov/geo), including 90 samples from GSE11969, 83 samples from GSE30219, 226 samples from GSE31210, 106 samples from GSE37745, 127 samples from GSE50081, 442 samples from GSE68465 and 105 samples from GSE81089, which were used as public validation cohorts. First, we log2-transformed the mRNA expressions of these public validation sets. After the quantiles were normalized, we selected the expression of genes with several probes as the mean expression.

### Sample analysis and quantitative real-time polymerase chain reaction (qRT-PCR)

Between May ‘13 and September ‘14, a total of 102 frozen and surgically resected tissue samples from patients with LUAD were collected from the First Affiliated Hospital of Zhengzhou University. The samples were snap-frozen promptly after surgical resection and stored in liquid nitrogen. We extracted the total RNA from the stored tissues using RNAiso Plus reagent (Takara, #9109) in accordance with the manufacturer’s instructions. Next, we reverse-transcribed the total RNA signature into single-stranded cDNA using the Prime Script™ RT reagent kit (Takara, #RR047A). The expression of the three selected genes in this prognostic signature was then detected via qRT-PCR. We used SYBR Premix Ex Taq II (Takara, #RR820A) reagent and Agilent Mx3005P software for data analysis. The three genes' expression values were first standardized to GAPDH and then log2 transformed for subsequent analysis. All primer sequences used for the three genes of interest, as well as GAPDH, are shown in Additional file 6: Table S1. The First Affiliated Hospital of Zhengzhou University’s Ethics Committee Board approved this protocol and monitored the study’s progress.

### Functional enrichment analysis and prognostic meta-analysis

We analyzed the gene ontology (GO) and Kyoto Encyclopedia of Genes and Genomes (KEGG) pathways to analyze the prognostic signature using DAVID 6.8 (http://david.abcc.ncifcrf.gov/home.jsp). Then, we used STATA software (version 12.0) to carry out a prognostic meta-analysis to better understand this signature's prognostic significance across different public cohorts. We used a random-effects model to calculate the pooled HR value.

### Immune cell infiltration analysis

The novel CIBERSORT method estimates cell fractions within complex tissues using gene expression levels in solid tumors. The results of this process stand in agreement with ground truth evaluation [19, 20]. The LM22 signature matrix included 547 genes that distinguished 22 immune cell phenotypes. These included distinct T- and B-cell subsets, as well as natural killer (NK) cells and other various myeloid subsets. We used CIBERSORT and the LM22 signature matrix to calculate the immune cell infiltration of patients with high- and low-risk disease statuses.

### Analysis of mutations, neoantigens, and expression of PD-L1 protein

We downloaded information on neoantigen and clonal neoantigen counts, mutation burden, and several subclonal neoantigens in patients with LAUD from the Cancer Immunome Atlas (TCIA) (https://tcia.at/home) [21]. We determined the PD-L1 protein expression based on a reverse-phase protein array (RPPA) analysis we obtained from the cbioPortal (http://www.cbioportal.org).

### Tumor Immune Dysfunction and Exclusion (TIDE) analysis

TIDE is an accurate computational framework to predict immunotherapeutic responses, especially to therapeutic strategies based on checkpoint blockade [22]. We integrated the mechanisms of primary tumor immune evasion (T-cell exclusion and dysfunction) into this novel method for modeling tumor immune evasion. The TIDE score is superior to many known biomarkers in predicting immunotherapy responses for melanoma and lung cancer, especially for patients who are treated with anti-PD-1/PD-L1 or anti-CTLA4 [22]. We uploaded the transcriptome profiles of patients from the TCGA cohort to the TIDE website (http://tide.dfci.harvard.edu), and after analyzing through the website, downloaded the scores for T-cell dysfunction and exclusion as well as the TIDE scores for all patients.

### Tumor Inflammation Signature analysis

Tumor Inflammation signature (TIS) is a novel clinical assay to predict immunotherapy responses in various tumors. We calculated the TIS scores for patients from the TCGA cohort according to the methods mentioned in the previous study [23].

### Signature generation and statistical analysis

We used a univariate Cox proportional hazards regression model to screen for genes that were strongly associated with OS in patients with LAUD. Then, we chose to use stepwise Cox proportional hazards regression analysis to reduce the total number of covariates and filter out the most valuable prognostic genes for the prediction of OS. A risk formula was constructed for each patient based on the expression of selected genes and corresponding regression coefficients from the stepwise Cox proportional hazards regression analysis. This information was used to classify patients into two groups: low risk and high risk. Next, we used a Kaplan–Meier analysis to determine the OS and RFS among the two risk groups. We used the Mann–Whitney U-test to explore estimates of immune cell infiltration, TMB load, the number of neoantigens (including clonal and subclonal neoantigens), expression of PD-L1 protein, the TIDE score, the T-cell dysfunction score, the T-cell exclusion scores, and TIS scores in the high- and low-risk patient subsets. Then, a Cox proportional hazards regression analysis was used to determine if the combined gene signature was capable of predicting prognoses dependently. We used R software version 3.5.1 (https://www.r-project.org) to analyze all data and generate the corresponding figures. A *P*-value < 0.05 indicated statistical significance. All statistical tests were two-tailed.

## Results

### Identification of prognostic novel immune checkpoint members in LUAD

**Fig. 1:**
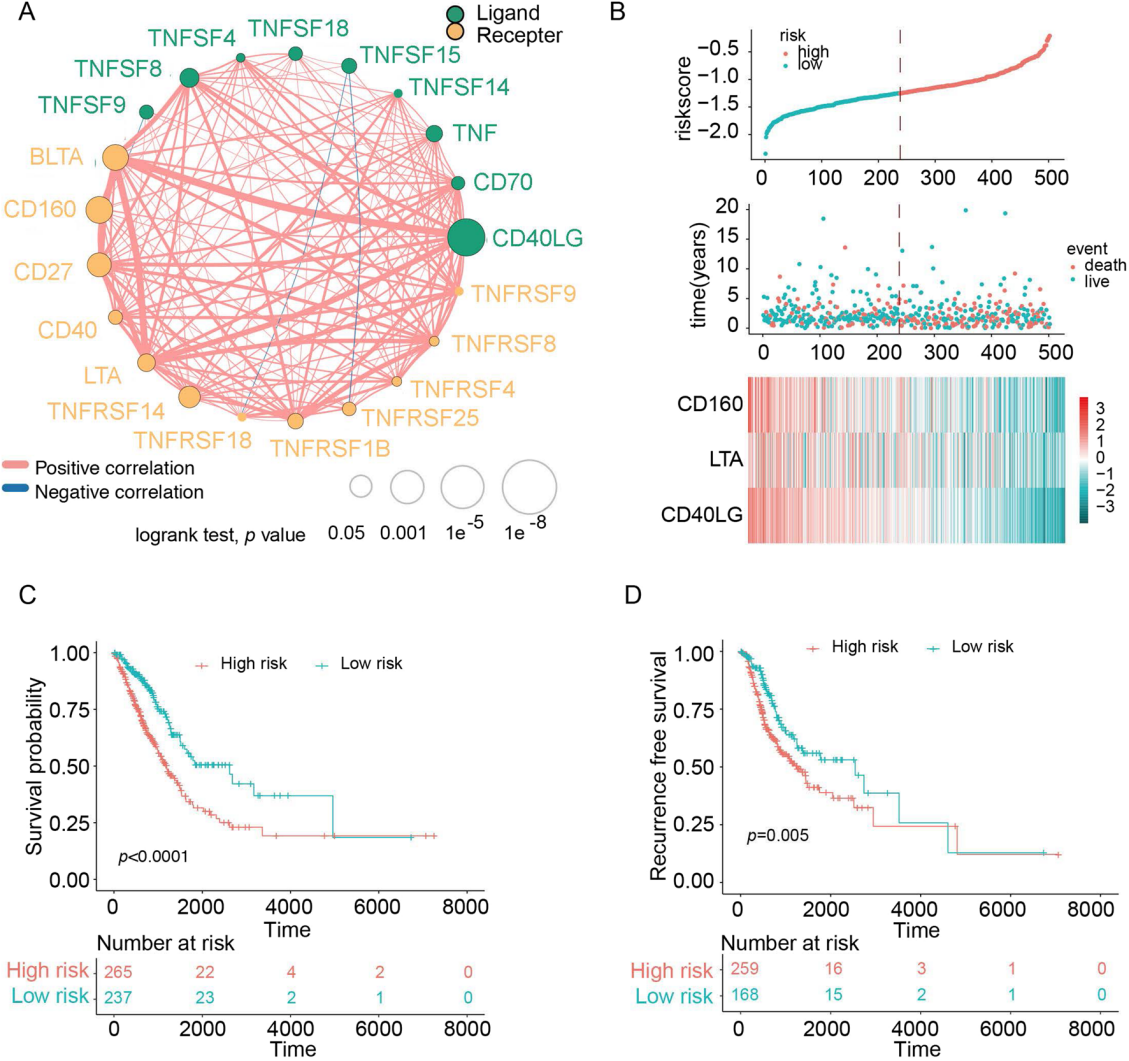
Generation of the novel immune checkpoint-based signature from a TCGA cohort. **A** Cellular interaction of novel immune checkpoints in LUAD. **B** Overview of risk score, survival outcomes, and gene expression distributions across included patients. **C** Kaplan–Meier curves of overall survival in all patients with LUAD, stratified by the novel immune checkpoint-based signature into a high- and low-risk group. **D** Kaplan–Meier curves of relapse-free survival in LUAD patients stratified by novel immune checkpoints-based risk signature

After a careful literature review, 21 clearly defined novel immune checkpoint members, which all belong to the TNF superfamily, were included in this study[15, 24–27]. We used a set of 502 patients with LUAD from the TCGA database as the training cohort for our model. The demographics of this cohort are listed in Table 1. The expression correlations among these 21 immune checkpoint members are displayed in Fig. 1A, which exhibited that many members have a strong positive association. The expression of the 21 defined members was explored in the training cohort. Then, a univariate Cox proportional regression analysis found that seven immune checkpoint genes were significantly associated with OS (*P* < 0.05, Additional file 6: Table S2). Interestingly, all seven genes (BTLA, CD160, CD27, CD40LG, LTA, TNFRSF14, and TNFSF8) with low hazard ratios (hazard ratio < 1) were positively correlated with favorable survival (Additional file 6: Table S2).

**Table 1:** Clinical characteristics of the patients from multiple institutions

Characteristics	TCGA n = 502	GSE11969 n = 90	GSE30219 n = 83	GSE31210 n = 226	GSE37745 n = 106	GSE50081 n = 127	GSE68465 n = 442	GSE81089 n = 105	Independent n = 102
Age, year									
≥ 60	356	58	46	130	64 42	108 19	314	87	53
< 60	136	32	37	96	42	19	128	18	49
NA	10	0	0	0	0	0	0	0	0
Sex									
Male	231	47	65	105	46	65	223	37	56
Female	271	43	18	121	60	62	219	68	46
Smoking history									
Yes	72	45	NA	111	NA	92	300	94	60
No	416	45	NA	115	NA	23	49	11	43
NA	14	0	NA	0	NA	12	93	0	
TNM stage									
I and II	389	65	81	226	89	127	371	78	79
III and IV	105	25	2	0	17	0	68	27	23
NA	8	0	0	0	0	0	3	0	0
OS state									
Alive	320	50	43	35	77	51	236	48	76
Death	182	40	40	191	29	76	206	57	26

*NA* not available, *OS* overall survival

### Generation of the prognostic signature in the TCGA cohort

The seven immune checkpoint genes with prognostic significance in univariate comparisons were used for further analysis. Next, we used a stepwise Cox proportional hazard regression model to determine which set of variables provided the most efficient estimate of patient prognoses. This procedure identified three genes of interest: CD160, LTA, and CD40LG. We established the risk score formula based on the expression of these three genes and their corresponding coefficients: risk score = 0.1379 × LTA—0.1525 × CD160- 0.2595 × CD40LG. Each patient’s risk score was calculated, and patients were categorized as high- or low-risk based on the optimal cutoff point (determine from the training data set, Fig. 1B). The high-risk group had a worse OS (*P* < 0.0001) and RFS (*P* = 0.0050) than the low-risk group (Fig. 1C, D). According to the multivariable Cox analysis, this novel risk score independently predicted OS in the TCGA cohort even after correcting for potential confounding from the other clinical variables (Table 2)

**Table 2:** Univariable and multivariable Cox regression analysis of the novel immune checkpoints-based signature and overall survival in TCGA cohort

	Univariable analysis			Multivariable analysis		
Variable	HR	95%CI	*P* value	HR	95%CI	*P* value
Age						
≥ 60 or < 60	1.0855	0.7524–1.5658	0.6609	1.3874	0.9432–2.0410	0.0963
Sex						
Male or female	1.1320	0.8254–1.5525	0.4419	0.9599	0.6849–1.3455	0.8125
Smoking history						
Yes or no	0.9332	0.6030–1.4444	0.7565	1.04711	0.6408–1.7110	0.8542
T stage						
1, 2, 3 or 4	1.9309	1.4885–2.5049	< 0.0001	1.1945	0.9648–1.4790	0.1029
Lymphatic metastasis						
Yes or no	1.0001	0.8281–1.2096	< 0.0001	1.2839	0.9116–1.8082	0.1526
TNM stage						
I, II, III or IV	1.5867	1.3685–1.8396	< 0.0001	1.3638	1.1063–1.6811	0.0037
EGFR status						
MUT or WT	1.4752	0.9718–2.2394	0.0679	1.4295	0.9034–2.2620	0.1270
KRAS status						
MUT or WT	1.2462	0.8864–1.7521	0.2054	1.3121	0.9148–1.8820	0.1397
Risk score						
High or low	2.5718	1.6286–4.0612	< 0.0001	2.5167	1.5408–4.1107	0.0002

*HR* hazard ratio, *CI* confidence interval

.

### Validating the prognostic signature in independent cohorts

**Fig. 2:**
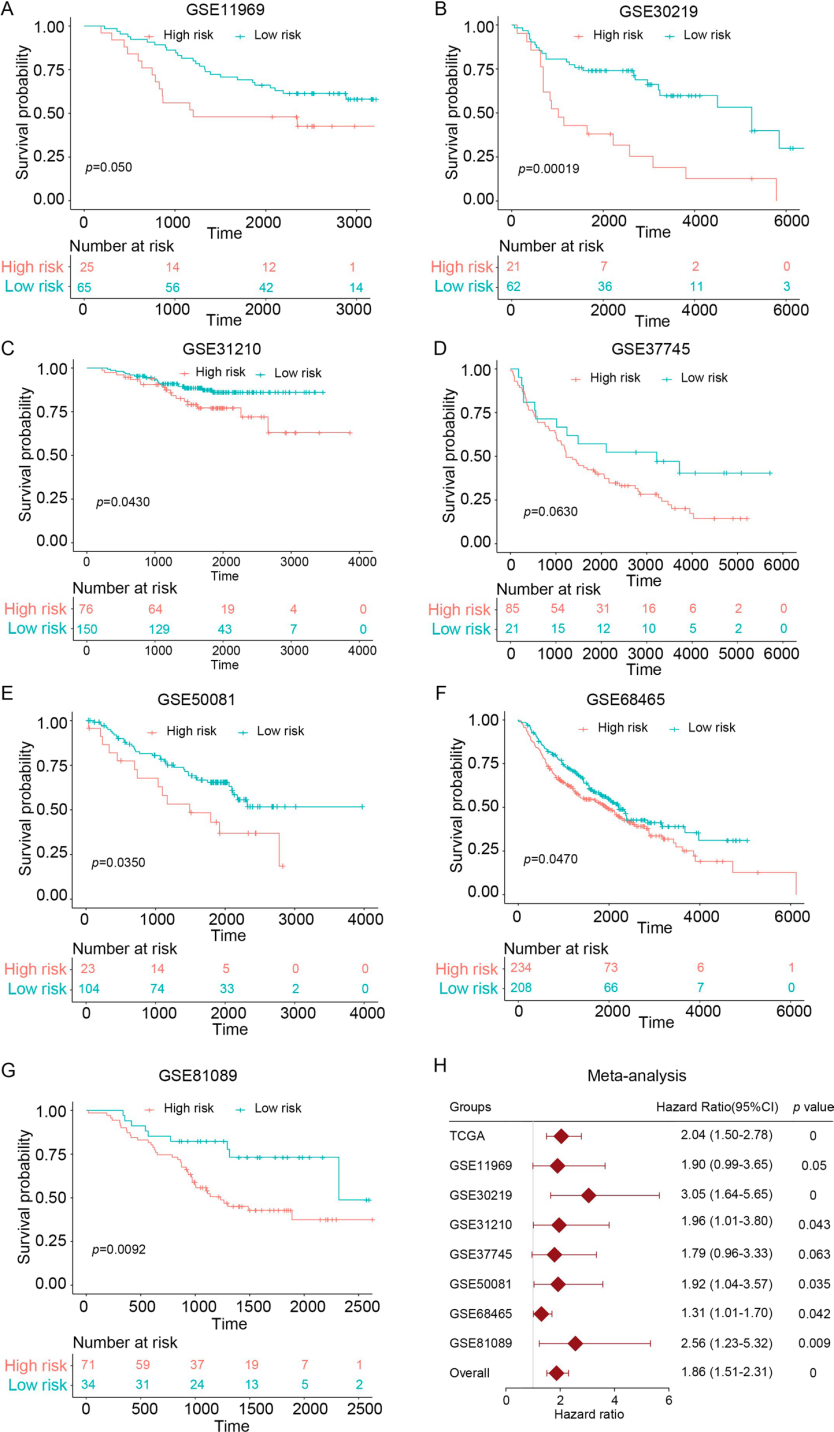
Validation of the novel immune checkpoint-based signature in patients with LUAD from GEO cohorts. **A**–**G** Kaplan–Meier curves of overall survival in the different GEO cohorts. **H** A meta-analysis of the prognostic value of the immune signature model when used to predict outcomes in the TCGA and GEO cohorts. The overall *P*-value (across all models) was determined from the meta-analysis

To test the prognostic signature's reproducibility in LAUD, we also applied this risk score formula in seven publicly available independent cohorts. Demographic data from these cohorts are presented in Table 1. All patients in these seven cohorts were divided into high- and low-risk groups based on the optimal risk-score cutoff that was determined from the training data set. Of interest, we found that patients in the high-risk group had an unfavorable OS than those in the low-risk group, especially in six of the test data sets: GSE11969 (*P* = 0.0496, Fig. 2A), GSE30219 (*P* = 0.00019, Fig. 2B), GSE31210 (*P* = 0.0430, Fig. 2C), GSE50081 (*P* = 0.0350, Fig. 2E), GSE68465 (*P* = 0.0470, Fig. 2F), GSE81089 (*P* = 0.0092, Fig. 2G). In the seventh group, stratification based on the most optimal risk cutoff only indicated a borderline difference between the OS in the high- and low-risk groups in the GSE37745 cohort (*P* = 0.0630, Fig. 2D). Furthermore, to validate the prognostic value of the novel immune checkpoints-based signature, we also performed a prognostic meta-analysis in these seven GEO cohorts and the TCGA cohort. The results of the analysis provide further evidence that this prognostic signature is a risk predictor in LAUD (*P* < 0.001) (Fig. 2H).

**Fig. 3:**
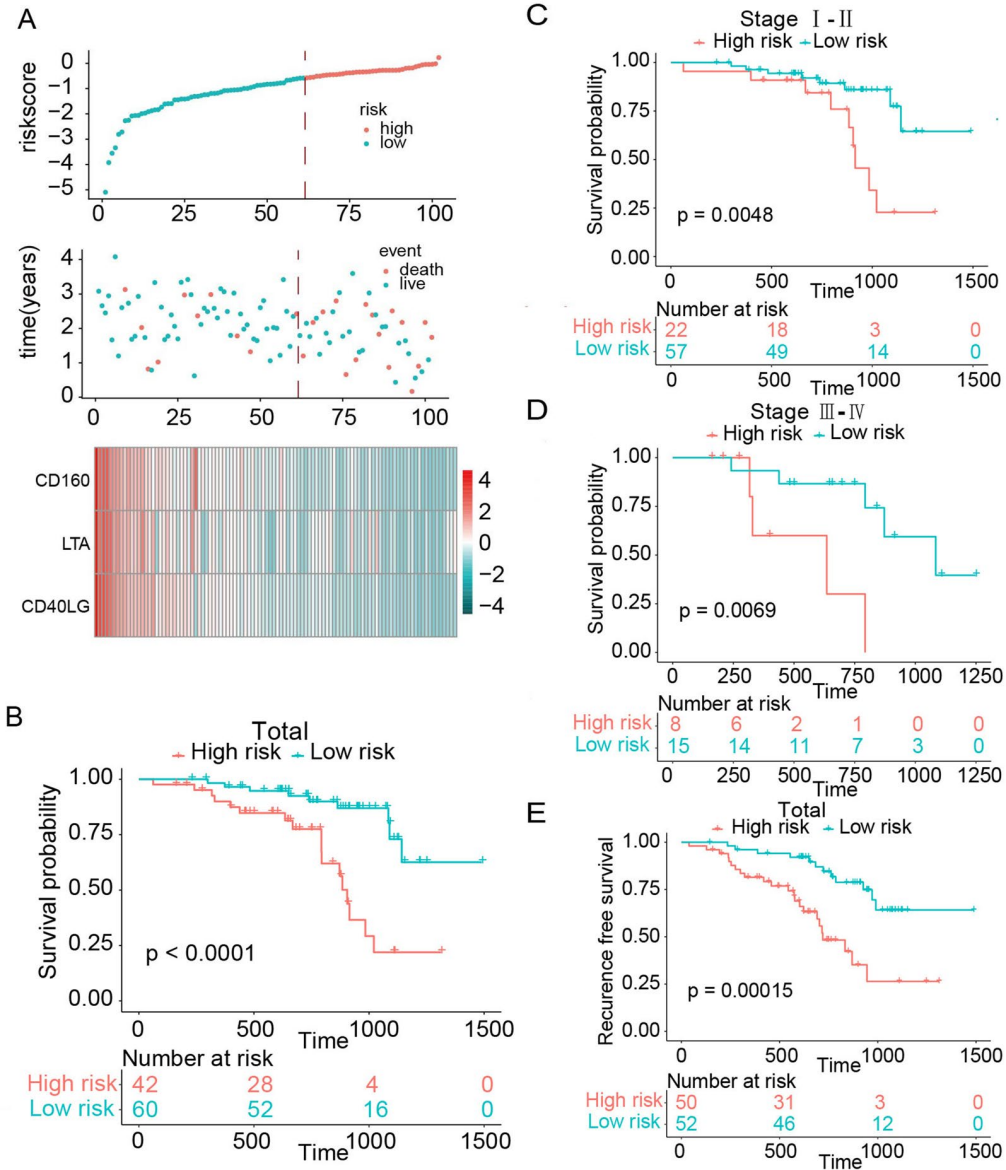
Validating the prognostic value of novel immune checkpoints-based signature in an independent cohort of 102 frozen tissue samples. **A** The distribution of risk score, survival status and gene expression panel. **B** Overall survival in all patients with LUAD, separated on the basis of risk scores (Kaplan–Meier analysis). **C** Kaplan–Meier curves of overall survival in patients with early-stage (stage I and II) LUAD, stratified by risk groups. **D** Kaplan–Meier curves of overall survival in patients with advanced-stage (stage III and IV) LUAD, stratified by risk score. **E** Kaplan–Meier curves of relapse-free survival in all patients with LUAD, stratified by the risk groups

To assess our signature’s applicability to clinical settings, we used qRT-PCR to validate this risk score in another independent cohort consisting of frozen samples from 102 patients with LUAD. This cohort’s demographic data are presented in Table 3. We used the risk score formula to divide the patients into two groups (Fig. 3A). We found a significant difference in OS (*P* < 0.0001) and RFS (*P* = 0.00015) between the two risk groups (Fig. 3B, E). Since LUAD patients with different clinical stages have different treatment principles and prognoses [28], we also applied this risk score within early- (clinical stage I and II) and advanced-stage (clinical stage III and IV) subgroups. We observed that patients in these clinical-stage subgroups who were at high risk had shorter survival than patients at low risk (Fig. 3C, D). Finally, we confirmed that this risk score was independently related to OS in these 102 patients with LAUD using qPCR data and multivariable Cox analysis (Table 3)

**Table 3:** Univariable and multivariable Cox regression analysis of the novel immune checkpoints-based signature and overall survival in independent cohort

	Univariable analysis			Multivariable analysis		
Variable	HR	95%CI	*P* value	HR	95%CI	*P* value
Age						
≥ 60 or < 60	1.0729	1.0205–1.1280	0.0060	1.1151	1.0474–1.1871	0.0006
Sex						
Male or female	1.1943	0.5473–2.6062	0.6556	1.0806	0.4709–2.4796	0.8548
Smoking history						
Yes or no	1.0080	0.4618–2.2002	0.9841	0.6269	0.2693–1.4593	0.2787
T stage						
1, 2, 3 or 4	1.2456	0.8004–1.9384	0.3305	0.8782	0.4234–1.8216	0.7272
Lymphatic metastasis						
Yes or no	4.2542	1.8319–9.8794	0.0008	3.4297	1.0293–11.4279	0.0447
TNM stage						
I, II, III or IV	2.4857	1.4564–4.2426	0.0060	1.8899	0.6834–5.2266	0.2200
Risk score						
High or low	3.0210	1.4103–6.4713	0.0044	3.1143	1.3997–6.9293	0.0054

*HR* hazard ratio, *CI* confidence interval

.

### Validation of the prognostic signature in important clinical subsets.

We next explored the association between prognosis and risk score for early- and advanced-stage samples in the TCGA cohort since the clinical stage is known to affect prognosis. For the early-stage subgroup, high-risk patients suffered shorter OS (*P* = 0.00012) and RFS (*P* = 0.0058) than their low-risk counterparts (Additional file 1: Fig. S1A, C). Similarly, in the advanced-stage subgroup, high-risk samples had a worse OS relative to low-risk ones (*P* = 0.17) (Additional file 1: Fig. S1B). However, among patients with advanced-stage disease, there was a borderline difference in RFS between the high- and low-risk groups (*P* = 0.071) (Additional file 1: Fig. S1D).

Patients above the risk cutoff suggested significantly inferior OS in other important clinical feature subtypes of the TCGA cohort, including older (age ≥ 60), younger (age < 60), male, female, and smoker relative to non-smokers (Additional file 2: Fig. S2). Next, considering the critical significance of the EGFR and KRAS mutations in LAUD, we further investigated the prognostic signature's performance in patient subgroups with EGFR and KRAS mutation status. Our prognostic signature accurately stratified varying OS for the EGFR wild-type (WT) vs. mutation (MUT), KRAS WT/MUT, or EGFR/KRAS WT subtypes (Additional file 3: Fig. S3). Last, we evaluated the risk scores across the three expression subtypes in LAUD: magnoid, squamoid, and bronchioid [29]. The risk score was also effective in stratifying patients with different OS in the magnoid and bronchioid subgroups, while showing a borderline difference in the squamiod subgroup (*P* = 0.0580) (Additional file 4: Fig. S4).

### Biological pathways and inflammatory responses analysis of the prognostic signature

**Fig. 4:**
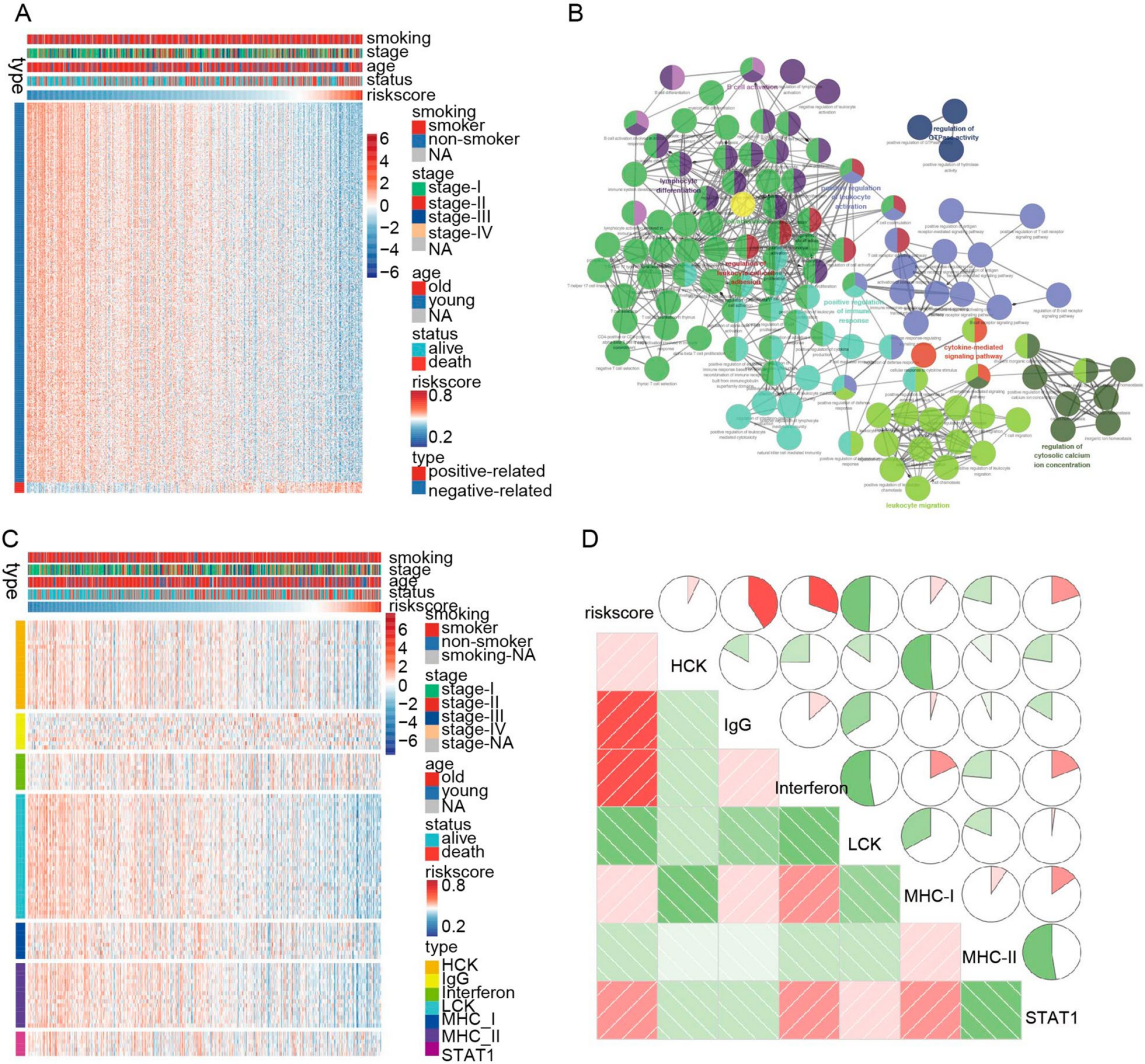
The prognostic signature-related biological pathways. **A** The primary genes in the novel immune checkpoint-based signature in patients with LUAD (Pearson |R|> 0.45). **B** GO and KEGG analysis of the genes. **C** The association between risk score and inflammatory activities in patients with LUAD. **D** corrgram of the prognostic signature and inflammatory metagenes

To explore this prognostic signature's related and potential biological pathways, we selected the genes that are strongly related to the signature (Pearson |R|> 0.45). The results demonstrated that about 436 genes were negatively, and 12 genes were positively associated with the risk score (Fig. 4A). Next, we performed GO and KEGG analysis for these genes, which showed that they are mainly enriched in immune response and leukocyte activation pathways (Fig. 4B). Meanwhile, we sought to further investigate the relevant inflammatory responses of the risk score. We analyzed the relationships between the seven clusters of metagenes identified from univariate (HCK, interferon, LCK, MHC-I, MHC-II, STAT1, IgG) and risk score [30]. The seven clusters of metagenes found here broadly represent pathways related to inflammatory responses and immune regulation. The profiles of these metagenes are shown in Fig. 4C. A Gene Sets Variation Analysis (GSVA) was performed to validate the seven metagene clusters [31]. The findings from this analysis indicated that the risk score was positively related to IgG, interferon, and STAT clusters, but negatively related to LCK and MHC-II clusters (Fig. 4D).

### Immune landscapes of the prognostic signature

**Fig. 5:**
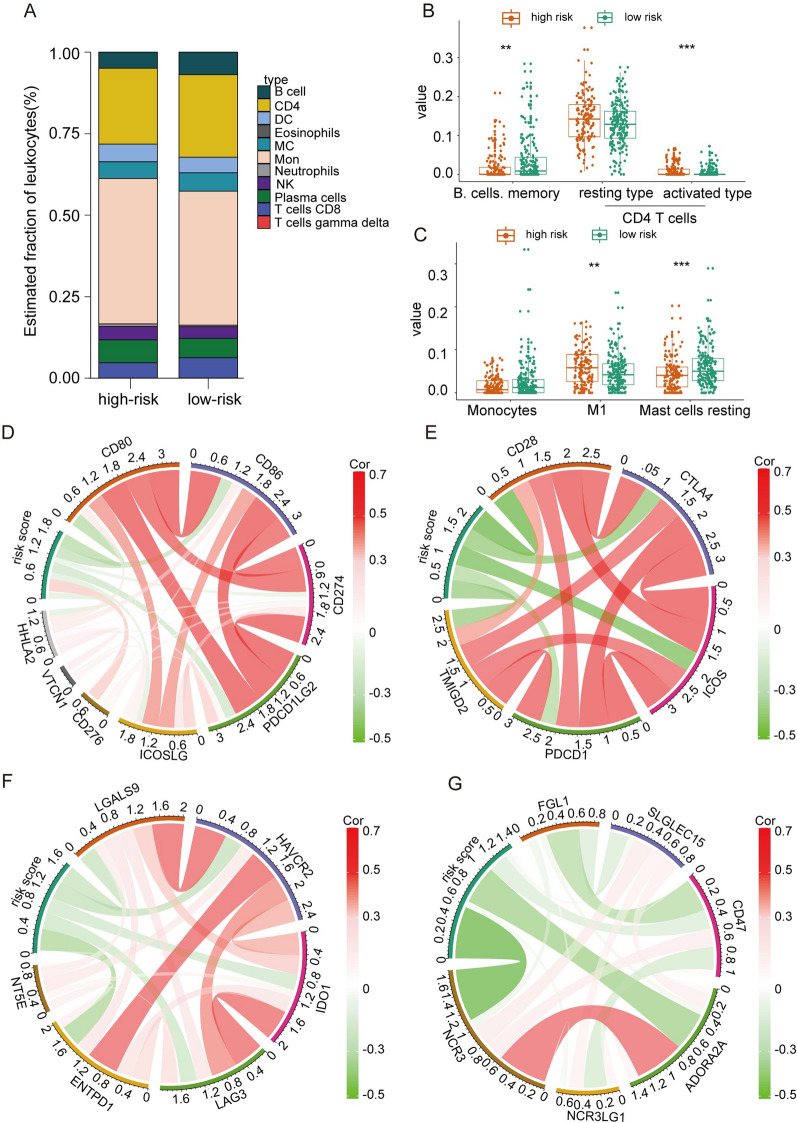
The immune landscape of the novel immune checkpoint-based signature in patients with LUAD. **A** Estimated immune cell expression proportion in high- and low-risk groups. **B** and **C** Detailed information on the different immune cell expression profiles across risk groups. **D**, **F**, **G**, and **H** The correlation between risk score and several critical immune checkpoints. *, **, and *** represent *P* < 0.05, *P* < 0.01, and *P* < 0.001, respectively

Since the prognostic signature is linked to immune response pathways, we intend to delve into the immune cell infiltration in patients with LUAD. Next, we combined CIBERSORT with LM22, a novel method for estimating 22 immune cell fractions and subtypes, to evaluate immune cell infiltration within the training cohort. We found that patients in the high and low-risk groups had distinct profiles of immune cells (Fig. 5A). The high-risk patient groups exhibited higher infiltration of resting CD4 T-cells, macrophagocyte M1, but lower infiltration of memory B-cells and resting T-cells (Fig. 5B, C). Meanwhile, because some immune checkpoints can construct a communication system to modulate antitumor immune responses [32], we next sought to explore the correlation between patient risk scores and several essential immune checkpoints. The Pearson correlation analysis suggested that the risk score was positively correlated with CD276. CD276, a vital immune checkpoint of the B7-28 family, and the immunotherapies targeting CD276 have achieved positive results in various tumors (Fig. 5D–G) [33]. This may indicate that patients at higher risk may benefit from treatment with immunotherapies that target CD276.

### Relationship between the prognostic signature and immunotherapy responses

**Fig. 6:**
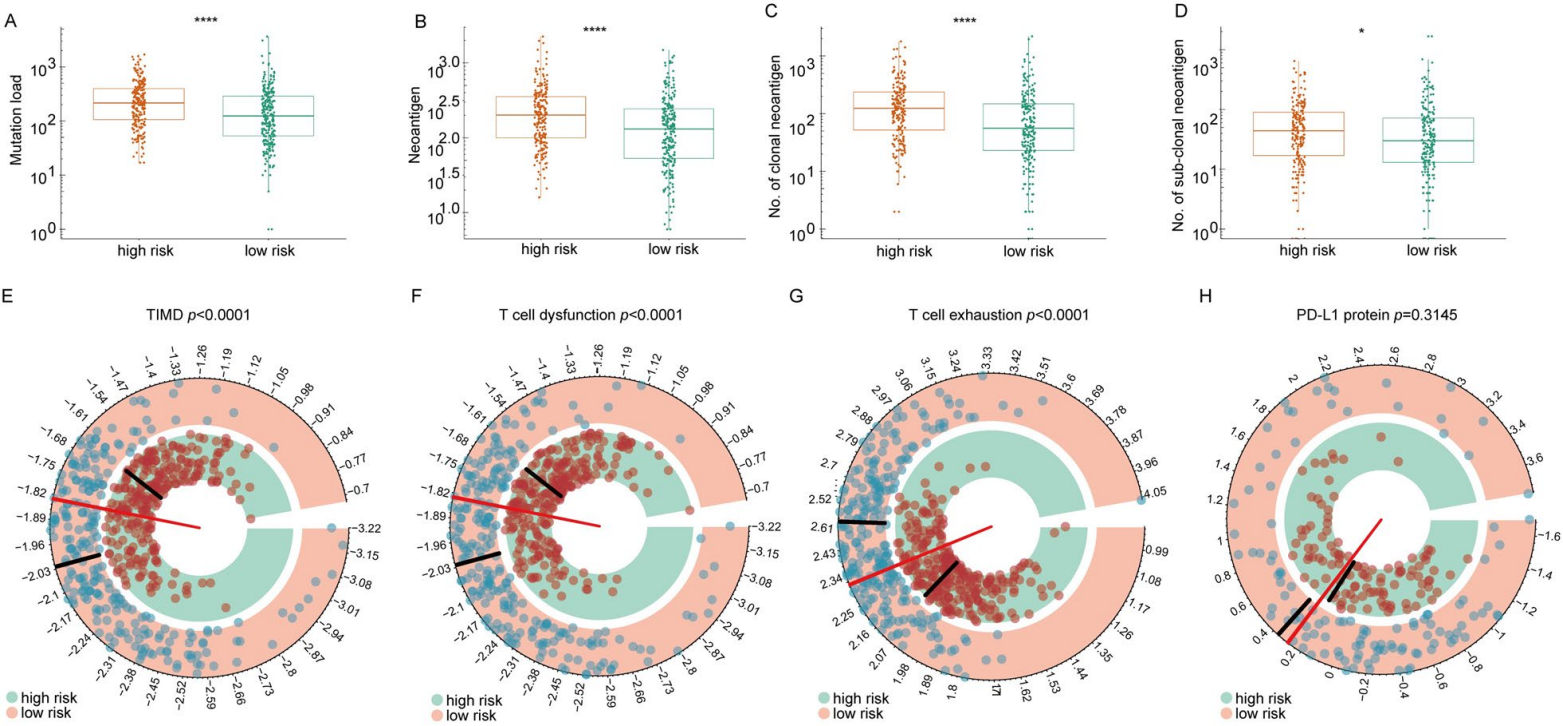
Distribution of immunotherapy response makers across risk groups.** A**, **B**, **C**, and **D** The distribution of TMB, number of neoantigens and clonal neoantigens, as well as the number of subclonal neoantigens across risk groups. **E** The distribution of PD-L1 expression across risk groups. **F** and **H** The distribution of TIDE score, T-cell exclusion score, and T-cell dysfunction score across risk groups. *, **, and *** represent *P* < 0.05, *P* < 0.01, and *P* < 0.001, respectively

Nowadays, immunotherapies targeting immune checkpoints are increasingly significant to cancer treatment and become the first-line choice in various tumors, especially for LAUD. Meanwhile, these novel immune checkpoint members also are potential and crucial candidate checkpoints for immunotherapies. We sought to find out the relationship between this signature and immunotherapy responses. We selected some classical and widespread biomarkers and analyzed their connection to the risk score [22, 32]. First, we calculated and evaluated the tumor mutation burden (TMB), the number of neoantigens, as well as the number of clonal and subclonal neoantigens among the high- and low-risk patients. The high-risk group exhibited higher TMB, and these three kinds of neoantigens (Fig. 6A–D). Second, we also compared the protein expression of PD-L1 in high- and low-risk patients and found a borderline, difference between the patients in the high- and low-risk groups in average PD-L1 protein expression levels (Fig. 6H). Finally, the TIDE score, an accurate and reliable biomarker for immunotherapy, was also applied in our analysis. We systematically explored the TIDE and T-cell dysfunction and exclusion scores in patients from different groups. As expected, high-risk patients exhibited lower TIDE scores and higher T-cell dysfunction and exclusion scores (Fig. 6E–G). However, the TIS score is negatively associated our risk scores, and the low-risk patients also demonstrated a higher TIS score than high-risk score counterparts, which contradicts with the above results (Additional file 5: Fig. S5). Together, these results indicated high-risk patients may be more likely to benefit from immunotherapies.

## Discussion

High-throughput sequencing and genomics research has ushered in the identification of emerging biomarkers and therapeutic targets. These advancements have also improved our understanding of tumors. However, there is little information on which biomarkers may predict immune therapy responses and prognoses, revealing tumor immune phenotypes in LAUD. To improve our understanding of the co-stimulatory signals important in LAUD, we combined three crucial novel immune checkpoints (*LTA*, *CD160*, and *CD40LG*) to create a novel signature. We used the TCGA database as a training cohort and systematically explored the association between the gene expressions of some novel immune checkpoint members and prognostic outcomes in patients with LUAD. In the process, we identified a novel immune checkpoints-based risk signature, which is closely related to OS and RFS of patients with LAUD. This novel risk signature was validated in seven different publicly available cohorts as well as 102 samples of frozen tumor tissues using qPCR data. The validity of our signature was further confirmed using a meta-analysis. Our results indicated that the risk signature was well-validated and significantly related to OS in various important clinical and mutation subgroups. Our novel signature independently predicted prognosis in patients with LAUD. We also explored and analyzed relevant mechanisms, immune predictors for immunotherapy, and immune cells infiltration of this risk signature. Thus, this signature may contribute to a deep and comprehensive understanding of precision immunotherapy for LAUD.

Currently, apart from B7-CD28 family members, there are several emerging and potential immune checkpoints for immunotherapy, such as some TNF superfamily members [16]. After analyzing the association between these novel immune checkpoints and prognoses in LUAD, we determined the most significant prognostic genes in these novel immune checkpoints, including protective (*LTA*) and risky (*CD160* and *CD40LG*) genes. Regarding the protective gene, *LTA*, a proinflammatory cytokine, belongs to the TNF superfamily, involved in the inflammatory and immune responses [34]. This molecule also can assist lymphocytes and stromal cells to induce cytotoxic effects on tumor cells [34, 35]. It was reported that the polymorphisms of *the LTA* gene are closely related to cancer risk, including LAUD and other adenocarcinoma malignancies [36]. Meanwhile, researchers indicated that depleting LTA-expressing lymphocytes with LTA-specific monoclonal antibodies may be useful in treating autoimmune diseases [37]. Also, in terms of risky genes, *CD160*—also known as *BY55*, is an immunoglobulin-like, glycosylphosphatidylinositol-anchored protein and expressed on natural killer cells, γδ T-cells, and a subset of CD4 + and CD8 + T-cells [38, 39]. CD160 was proven to bind to herpesvirus entry mediator (HEVM) with high affinity, which induces robust natural cells effector activity and suppressed T-cell responses in vitro [40, 41]. B and T lymphocyte attenuator (BTLA) is a novel checkpoint receptor for immunotherapy, while CD160 shares the same ligands with it as BTLA repaired receptor, suggesting that CD160 inhibitory also may be a promising target for immunotherapy [27]. CD40LG is the ligand of CD40, and after they get combined, it will stimulate proinflammatory gene expression, including interleukins (IL)-1, IL-6, IL-8, IL-12, TNF-α, IFN-γ, and monocytic chemoattractant protein (MCP)-1. The activation of the CD40/CD40LG system is a major contributor to carcinogenesis. When CD40LG antibodies are used to disrupt the CD40/CD4OLG system’s function, it leads to the suppression of tumor cells [42]. However, some researchers demonstrated that the membrane-stable CD40LG mutant gene transfer into the CD40-positive LUAD cell line. This gene transfer reduces cell proliferation and enhances apoptosis [43]. Thus, the role of CD40LG in LUAD is still uncertain and requires further in-depth study and exploration. Similarly, the functions of LTA and CD160 in LUAD are unclear, and more relevant research are urgently needed.

We also investigated the potential genetic mechanisms of this signature. Risk-score-related genes were mainly enriched in immune response and leukocyte activation processes and pathways after correlation analysis. We analyzed seven immune-related metagenes to better understand the relationship between risk signature and immune responses. We found that risk score was negatively associated with LCK and MHC_II clusters, suggesting that high-risk patients have decreased B-cell function and impaired antigen-presentation ability. We also found higher infiltration of resting type CD4 + T-cells in high-risk patients, suggestive of immunosuppression. Next, we found that the patient risk score was positively associated with the expression of CD276. CD276 is a crucial immune checkpoint member of the B7-28 family that plays a pivotal role in inhibiting T-cell function, and immunotherapies that target CD276 have achieved positive results in various tumors [33]. This showed that patients at elevated risk may benefit from immunotherapies that target CD276. We also found that patients at elevated risk exhibited higher average TMB levels, more T-cell dysfunction, greater exclusion scores, lower TIDE scores. Of interest, despite this trend, PD-L1 protein expression differed across risk groups. PD-L1 and TMB are currently the most reliable biomarkers for predicting responses to PD-1/PD-L1 immunotherapy [44, 45]. These findings suggest that high-risk patients are mostly immunosuppressed and are therefore better candidates for immunotherapy.

Although this signature was successfully validated across multiple cohorts and appeared to act as an independent prognostic factor for LUAD, this study had several limitations. Firstly, it was a retrospective study that should be validated in prospective, large-scale cohorts. Secondly, we only examined some novel immune checkpoint genes, which may limit our signature's predictive capacity. However, this new classifier also provides more information on the state of the TME. Finally, none of the patients in this study underwent immunotherapy, thus our prediction of immunotherapy responsiveness is merely theoretical. Future studies should address these limitations.

In conclusion, we found that analyzing tumor expression of LTA, CD160, and CD40LG represents a novel immune signature that may be useful for predicting prognosis and response to immunotherapy in patients with LUAD. Further validation of these findings may improve the ability to shed some light on screening appropriate patients for are most likely to benefit from immunotherapy, enabling increasingly personalized, evidence-based care.

## Supplementary Information

**Additional file 1: Fig S1**. Validation of the prognostic predictive capacity of novel immune checkpoint-based signature in clinical subgroups. (A) and (B) Kaplan-Meier curves of overall survival in patients with early-stage (stage I and II) and advanced-stage (stage III and IV) LUAD based on the risk score. (C) and (D) Kaplan-Meier curves of relapse-free survival in patients with early- (stage I and II) and advanced-stage (stage III and IV) LUAD based on the risk score.

**Additional file 2: Fig S2**. Validation of the prognostic performance of the novel immune checkpoints-based signature across clinical subgroups. Kaplan-Meier curves of overall survival in male (A), female (B), older (C), younger (D), smokers (E), and non-smokers (F), separated on the basis of risk score.

**Additional file 3: Fig S3**. Validation of the prognostic performance of the novel immune checkpoints-based signature in different mutation status. Kaplan-Meier curves of overall survival in patients carrying EGFR-WT (A), EGFR-MUT (B), KRAS-WT (C), KRAS-MUT (D) and EGFR/KRAS-WT (E) based on the risk score.

**Additional file 4: Fig S4**. Validation of the prognostic performance of the immune checkpoints-based signature in different molecular subtypes. (A) The distribution of risk score in in bronchioid, magnoid and squamiod subtypes. (B), (C) and (D) Kaplan-Meier curves of overall survival based on risk score in bronchioid, magnoid and squamiod subtypes. *, **, and *** represent P < 0.05, P < 0.01, and P < 0.001, respectively.

**Additional file 5: Fig S5**. The relationship between TIS score and our novel signature. (A) The distribution of TIS scores across risk groups. (B) The correlation between TIS scores and our risk scores in the TCGA cohort.

**Additional file 6: Table S1**. Primer Sequences for q-PCR. **Table S2**. Univariate Cox proportional regression analysis of the valued prognostic genes in TCGA cohort.

## Data Availability

Most of the data sets used and/or analyzed during the current study are publicly available data from TCGA and Gene Expression Omnibus (GEO) databases (GSE11969, GSE30219, GSE31210, GSE37745, GSE50081, GSE68645, and GSE81089). All data of the independent cohort in the current study were available from the corresponding authors in a reasonable request.

## Abbreviations

AUC: Area under the curve
CI: Confidence interval
GEO: Gene Expression Omnibus
GO: Gene Oncology
HR: Hazard ratio
KEGG: Kyoto Encyclopedia of Genes and Genomes
LUAD: Lung adenocarcinoma
LUSC: Lung squamous cell carcinoma
NA: Not available
NSCLC: Non–small cell lung cancer
OS: Overall survival
PD-1: Programmed death 1 receptor
PD-L1: Programmed death-ligand 1
qRT-PCR: Quantitative real-time polymerase chain reaction
RFS: Recurrence-free survival
ROC: Receiver operating characteristic
TCGA: The Cancer Genome Atlas
TIDE: Tumor Immune Dysfunction and Exclusion analysis
TKIs: Tyrosine kinase inhibitors
TMB: Tumor mutation burden
TME: Tumor microenvironment
TNF: Tumor necrosis factor
TNFSF: TNF ligands superfamily
TNFRSF: TNF receptors superfamily
