# Supplementary figures and images for: A novel immune checkpoints-based signature to predict prognosis and response to immunotherapy in lung adenocarcinoma
Source: J Transl Med. 2022 Jul 25;20:332. doi: 10.1186/s12967-022-03520-6 (PMC9310422; doi:10.1186/s12967-022-03520-6)

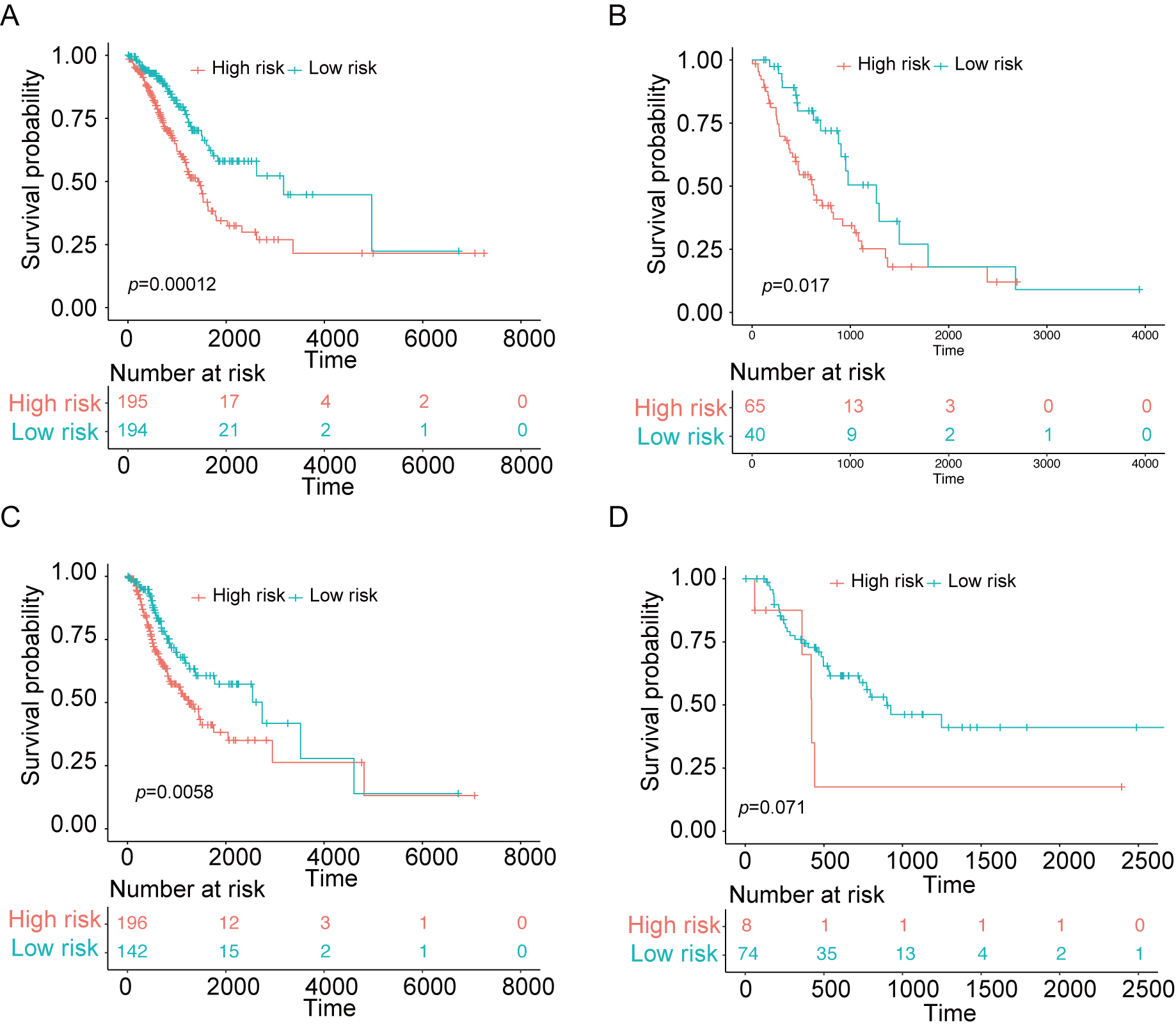

Supplement: Supplementary file 1 — Additional file 1: Fig S1. Validation of the prognostic predictive capacity of novel immune checkpoint-based signature in clinical subgroups. (A) and (B) Kaplan-Meier curves of overall survival in patients with early-stage (stage I and II) and advanced-stage (stage III and IV) LUAD based on the risk score. (C) and (D) Kaplan-Meier curves of relapse-free survival in patients with early- (stage I and II) and advanced-stage (stage III and IV) LUAD based on the risk score. [file 12967_2022_3520_MOESM1_ESM.tif]

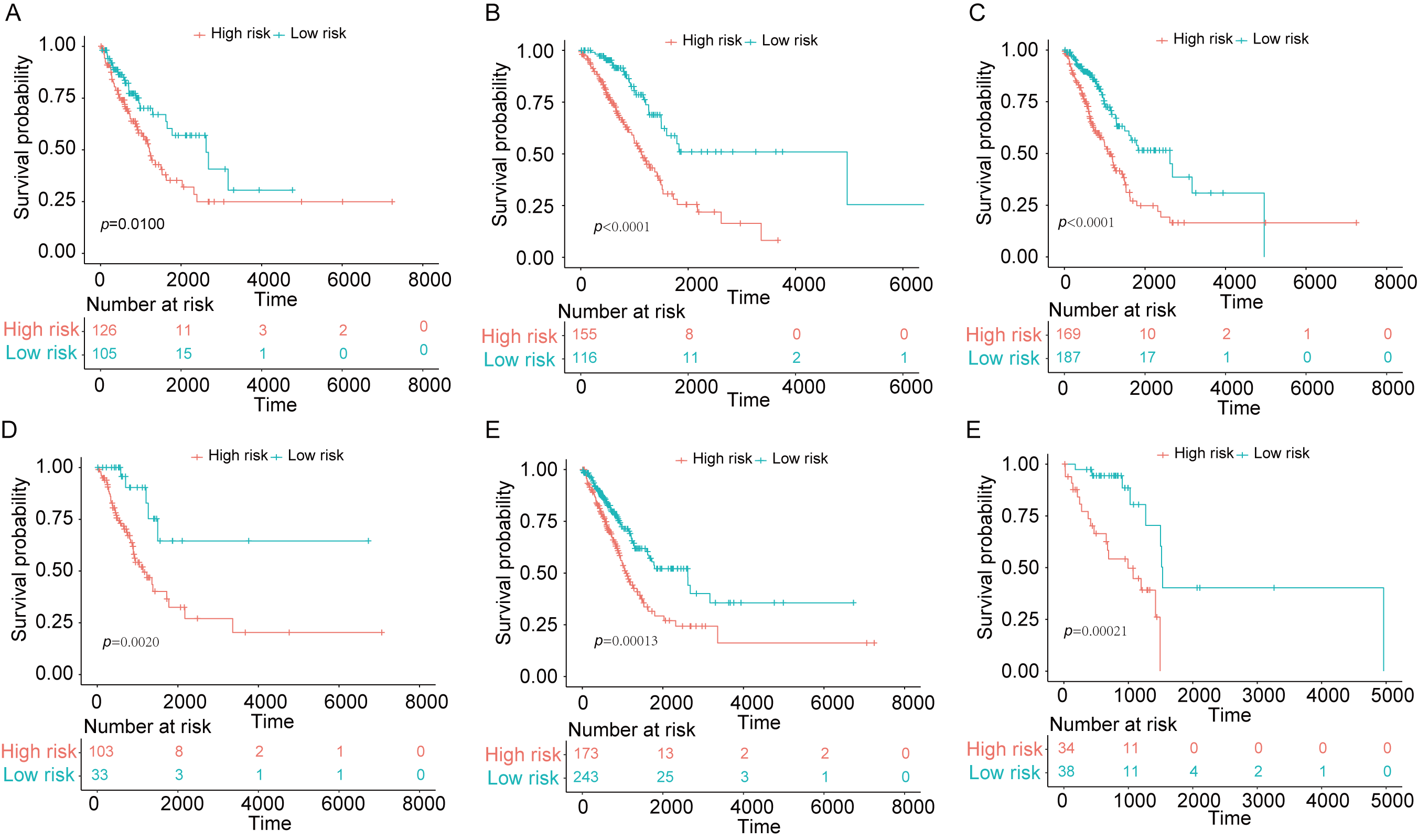

Supplement: Supplementary file 2 — Additional file 2: Fig S2. Validation of the prognostic performance of the novel immune checkpoints-based signature across clinical subgroups. Kaplan-Meier curves of overall survival in male (A), female (B), older (C), younger (D), smokers (E), and non-smokers (F), separated on the basis of risk score. [file 12967_2022_3520_MOESM2_ESM.tif]

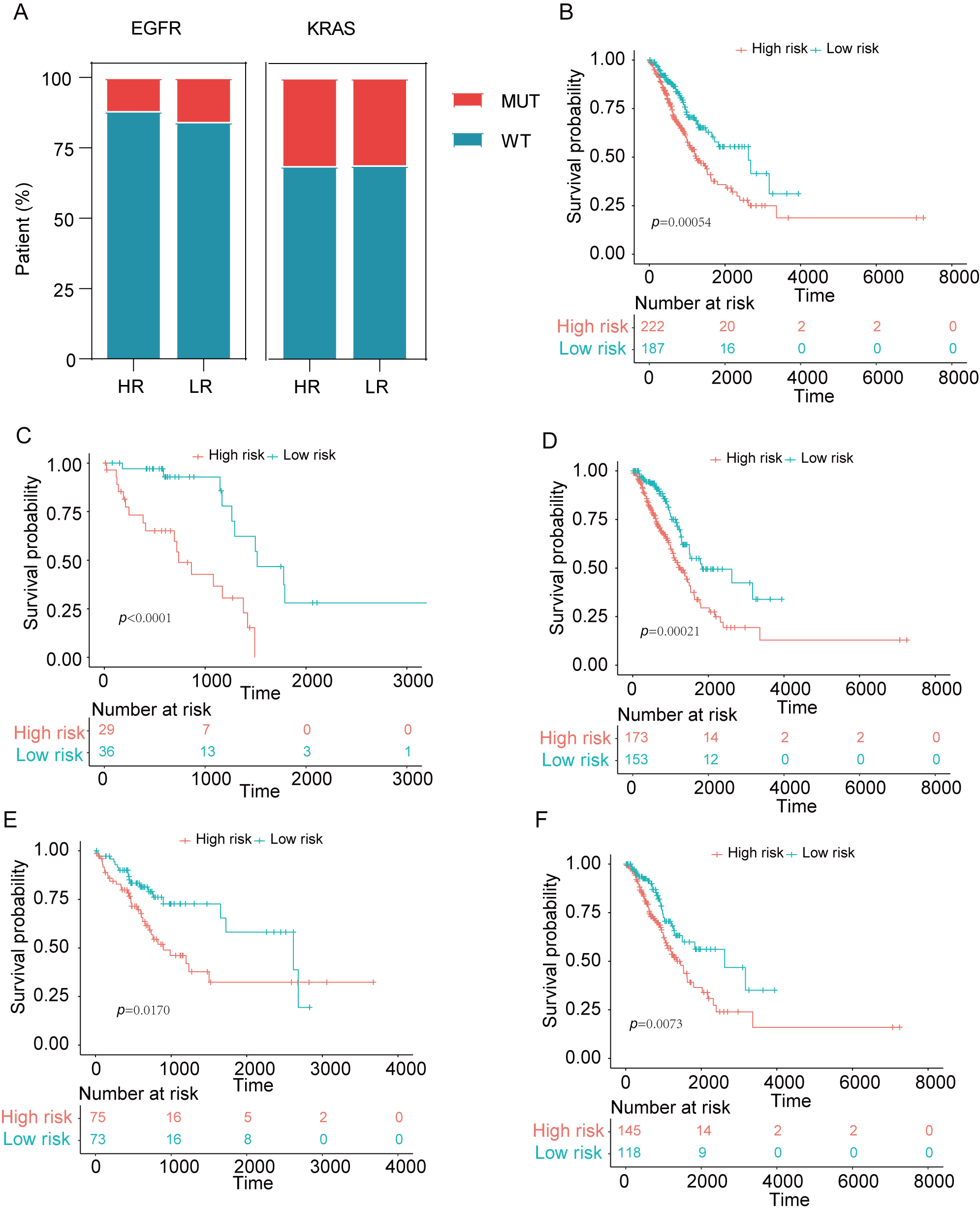

Supplement: Supplementary file 3 — Additional file 3: Fig S3. Validation of the prognostic performance of the novel immune checkpoints-based signature in different mutation status. Kaplan-Meier curves of overall survival in patients carrying EGFR-WT (A), EGFR-MUT (B), KRAS-WT (C), KRAS-MUT (D) and EGFR/KRAS-WT (E) based on the risk score. [file 12967_2022_3520_MOESM3_ESM.tif]

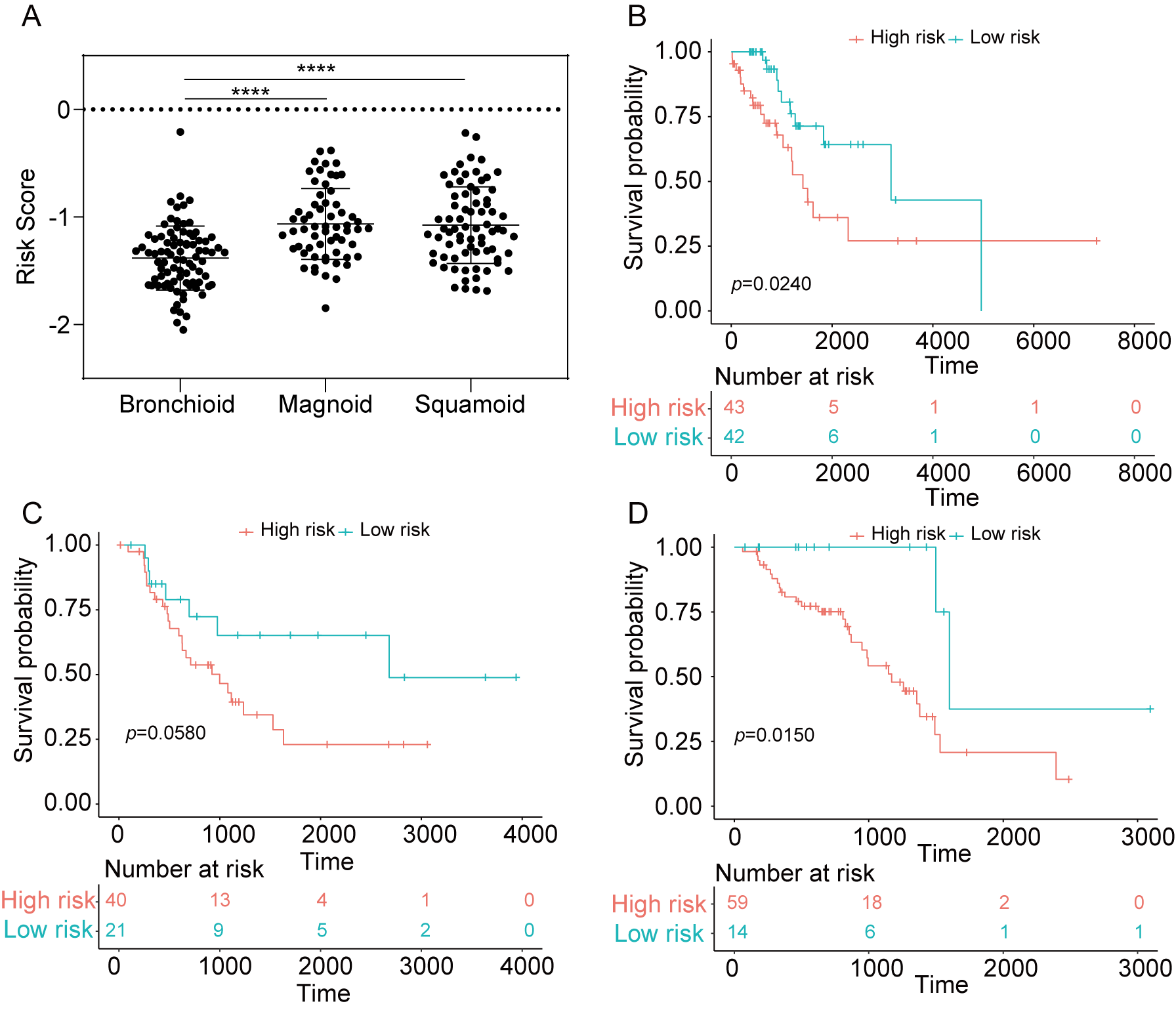

Supplement: Supplementary file 4 — Additional file 4: Fig S4. Validation of the prognostic performance of the immune checkpoints-based signature in different molecular subtypes. (A) The distribution of risk score in in bronchioid, magnoid and squamiod subtypes. (B), (C) and (D) Kaplan-Meier curves of overall survival based on risk score in bronchioid, magnoid and squamiod subtypes. *, **, and *** represent P < 0.05, P < 0.01, and P < 0.001, respectively. [file 12967_2022_3520_MOESM4_ESM.tif]

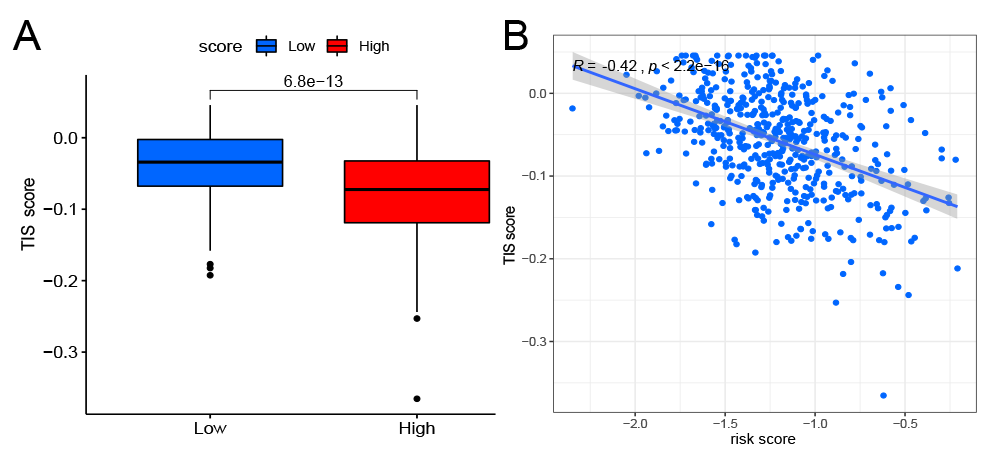

Supplement: Supplementary file 5 — Additional file 5: Fig S5. The relationship between TIS score and our novel signature. (A) The distribution of TIS scores across risk groups. (B) The correlation between TIS scores and our risk scores in the TCGA cohort. [file 12967_2022_3520_MOESM5_ESM.tif]
