# Supplementary material for: A novel immune checkpoints-based signature to predict prognosis and response to immunotherapy in lung adenocarcinoma
Source: J Transl Med. 2022 Jul 25;20:332. doi: 10.1186/s12967-022-03520-6 (PMC9310422; doi:10.1186/s12967-022-03520-6)
Supplement: Supplementary file 6 — Additional file 6: Table S1. Primer Sequences for q-PCR. Table S2. Univariate Cox proportional regression analysis of the valued prognostic genes in TCGA cohort. [file 12967_2022_3520_MOESM6_ESM.docx]

Supplementary table 1. Primer Sequences for q-PCR.

| Gene Name | Forward Primer | Reverse Primer |
| --- | --- | --- |
| *CD40LG* | 5'-ACATACAACCAAACTTCTCCCCG-3' | 5'-GCAAAAAGTGCTGACCCAATCA-3' |
| *CD160* | 5'-GCTGAGGGGTTTGTAGTGTTT-3' | 5'-GTGTGACTTGGCTTATGGTGA-3' |
| *LTA* | 5'-CATCTACTTCGTCTACTCCCAGG -3' | 5'-CCCCGTGGTACATCGAGTG-3' |
| *GAPDH* | 5'-TCCAAGGATTGTGGTTATGGAGA-3' | 5'-AGCACAGGTGATGCAACTCTG-3' |

Supplementary table 2. Univariate Cox proportional regression analysis of the valued prognostic genes in TCGA cohort.

|  | Univariable analysis | | |
| --- | --- | --- | --- |
| Gene symbol | HR | 95%CI | *P* value |
| *BTLA* | 0.8498 | 0.7630-0.9465 | 0.0031 |
| *CD160* | 0.7899 | 0.6791-0.9188 | 0.0022 |
| *CD27* | 0.8682 | 0.7858-0.9592 | 0.0055 |
| *CD40LG* | 0.8194 | 0.7421-0.9049 | <0.0001 |
| *LTA* | 0.8918 | 0.8002-0.9939 | 0.0385 |
| *TNFRSF14* | 0.8253 | 0.7112-0.9577 | 0.0114 |
| *TNFSF8* | 0.8864 | 0.7979-0.9848 | 0.0247 |

Abbreviations: HR, hazard ratio; CI, confidence interval.
